# Changes in mental health service utilization before and during the COVID-19 pandemic: a nationwide database analysis in Korea

**DOI:** 10.4178/epih.e2023022

**Published:** 2023-02-14

**Authors:** Kyoung Hoon Kim, Sang Min Lee, Minha Hong, Kyu-Man Han, Jong-Woo Paik

**Affiliations:** 1 Health Insurance Review and Assessment Service, Wonju, Korea; 2 Department of Psychiatry, Kyung Hee University Hospital, Kyung Hee University School of Medicine, Seoul, Korea; 3 Department of Psychiatry, Myongji Hospital, Hanyang University College of Medicine, Seoul, Korea; 4 UNC Neuroscience Center, University of North Carolina, Chapel Hill, NC, USA; 5 Department of Psychiatry, Korea University College of Medicine, Seoul, Korea

**Keywords:** COVID-19, Mental health, Utilization, Interrupted time series analysis, Pandemics

## Abstract

**OBJECTIVES:**

The present study examined the impact of the coronavirus disease 2019 (COVID-19) pandemic on mental health service utilization through a comparative analysis of nationwide data regarding inpatient care users, outpatient visits, emergency department (ED) visits, and admissions via the ED before and during the pandemic.

**METHODS:**

Data from approximately 350,000 Koreans diagnosed with mental illness were analyzed in terms of hospitalization, outpatient visits, and ED visits between January 2018 and June 2021. An interrupted time series analysis was conducted to determine the significance of changes in mental health service utilization indicators.

**RESULTS:**

The number of hospital admissions per patient decreased by 1.2% at the start of the pandemic and 0.7% afterward. The length of hospital stay increased by 1.8% at the outbreak of the pandemic, and then decreased by 20.2%. Although the number of outpatients increased, the number of outpatient visits per patient decreased; the number of outpatient visits for schizophrenia (3.4%) and bipolar disorder (3.5%) significantly decreased immediately post-outbreak. The number of ED visits per patient decreased both immediately post-outbreak and afterward, and ED visits for schizophrenia (19.2%), bipolar disorder (22.3%), and depression (17.4%) decreased significantly immediately post-outbreak. Admissions via the ED did not show a significant change immediately post-outbreak.

**CONCLUSIONS:**

Mental health service utilization increased during the pandemic, but medical service use decreased overall, with a particularly significant decrease in ED utilization. As the pandemic worsened, the decline in outpatient visits became more pronounced among those with severe mental illness.

## GRAPHICAL ABSTRACT

[Fig f3-epih-45-e2023022]

## INTRODUCTION

The coronavirus disease 2019 (COVID-19) pandemic (hereinafter, the pandemic) has had a severe impact on individuals’ mental health [1]; community health surveys in various countries have shown an increase in anxiety, depression, stress, risk of suicide, and post-traumatic stress [2,3]. A meta-analysis also confirmed that depression, anxiety, sleep disorders, and psychological distress increased during the pandemic [4]. However, there is a lack of evidence on whether the worsening of these mental health indicators leads to the onset of mental disorders.

Healthcare system reforms in response to the pandemic have reduced the accessibility of health services and led to a decline in overall health service utilization and acute care due to fears of infection [5,6]. In terms of mental health service utilization, a German study reported decreased hospitalizations and a change in the diagnostic spectrum [7]. A nationwide study in the United States analyzed mental health service utilization before and during the pandemic among 190 million emergency department (ED) visits; it revealed that mental health issues, suicide attempts, and domestic violence cases decreased during the first few weeks of the pandemic, but subsequently increased to a level higher than the previous year [8]. Furthermore, data on pediatric hospital admissions in the United States showed a sharp decrease in hospital admissions for mental health issues during the first 3 months of the pandemic [9]. In a study that conducted a trend analysis of the entire population in Ontario, Canada, the results showed that hospital admissions and ED visits for mental health issues decreased by 30% and 37%, respectively, during the early stage of the pandemic; however, these figures returned to pre-COVID-19 levels 1 year after the outbreak of the pandemic. While there were concerns about the public health system becoming overburdened after recovery from the pandemic due to patients being unable to receive proper treatment or diagnosis during the pandemic, the Canadian study showed no increase in health service utilization.

In Korea, mass infections in psychiatric hospitals occurred repeatedly during the early stage of the pandemic, with psychiatric patients accounting for the highest number of COVID-19-related deaths [10]. However, the continued utilization of health services during the COVID-19 pandemic was possible in Korea because of adequate social distancing measures, unlike other countries that stopped providing these services. Korea experienced 3 waves of the pandemic in 2020. The third wave occurred throughout Korea, starting from November 2020 to January 2021; starting in January 2021, stricter social distancing measures were implemented throughout the country [11].

The present study investigated changes in mental health-related hospital admissions, outpatient visits, and admissions via the ED among all Koreans before and during the pandemic and analyzed the utilization of these services based on major mental disorders. The findings are intended to reveal real-world data based on a nationwide database and offer evidence for establishing mental health countermeasures against the pandemic.

## MATERIALS AND METHODS

### Data source and study population

This cross-sectional study used the International Classification of Disease, 10th edition to code and classify data. It investigated patients’ mental health service utilization by hospital admissions, outpatient visits, and admissions via the ED for primary diagnostic codes F.xx between January 2018 and June 2021. Psychiatric patients’ data were extracted from the National Health Insurance Claims Database (NHICD) managed by the Health Insurance Review and Assessment Service. The national health insurance system covers over 97% of the entire Korean population; thus, the database based on this system may be considered representative of the rate of health service utilization by all Koreans [12]. The NHICD was established for the payment of medical fees and includes detailed service information, such as patients’ demographic characteristics, diagnostic codes, prescriptions, and examinations.

### Outcome measures and variables

The number of hospital admissions, length of hospital stay, number of admissions via the ED, number of outpatient visits, and number of ED visits per patient between January 2018 and June 2021 were defined as health service utilization indicators. These indicators were measured on a quarterly basis, based on the dates of admissions, outpatient visits, and ED visits. ED visits were defined as cases in which the NHICD indicated admission through the ED or a free code for emergency medical service. The patients’ age groups were categorized as <20 years, 20-44 years, 45-64 years, 65-79 years, and ≥ 80 years. Mental disorders were classified as dementia (F01-F03), schizophrenia (F20-F29), bipolar disorder (F30-F31), depression (F32-F39), anxiety disorder (F40-F41), other mental disorders due to brain damage, dysfunction, and physical disease (F06), sleep disorders (F51), and “others” based on the disorders that appeared most frequently during the study period.

### Statistical analysis

Patients’ demographic characteristics by year were presented as descriptive statistics, which included frequencies, percentages, and means. Differences in the demographic characteristics were tested using analysis of variance for continuous variables and the chi-square test for categorical variables. The first COVID-19 wave in Korea began in April 2020; health service utilization before the pandemic (January 2018 to March 2020) and during the pandemic (April 2020 to June 2021) was analyzed. Quarterly health service utilization indicators were derived and used to analyze the effects of the pandemic. Further, interrupted time series analysis was performed to identify the significance of changes in the indicators for the overall trend (time), Q1 of 2020 when the pandemic began (intervention), and trends after the outbreak of the pandemic (time after intervention). For interpretation, the results of regression coefficients are given as percentages, which are defined as the mean value of the dependent variables divided by the regression coefficients and multiplied by 100. All statistical analyses were performed with SAS Enterprise Guide 7.1 (SAS Institute Inc., Cary, NC, USA), and an α-level less than 0.05 was considered significant.

### Ethics statement

This study was approved by the Institutional Review Board (IRB) of Hanyang University Myongji Hospital (IRB No. 2022-04-029). The requirement for informed consent was waived by the IRB because of the public nature of the NHICD data, where information is gathered by ID number but is not identifiable.

## RESULTS

### Mental health service use from January 2018 to June 2021

There was an increase in the number of patients who received treatment for mental illness from before the pandemic (n=3,319,429 in 2018 and n= 3,537,643 in 2019) to during the pandemic (n= 3,636,506 in 2020). While the number of individuals who received inpatient care decreased, the number of outpatients increased. With respect to age, those aged 20-44 years and ≥ 80 years showed a decrease in hospital admissions, but an increase in outpatient visits, whereas those aged 45-79 years showed a decrease in both hospital admissions and outpatient visits. Furthermore, both male and female patients showed a decrease in hospital admissions but an increase in outpatient visits. The trends of hospital admission and outpatient visits among health insurance subscribers and Medical Aid recipients were consistent with the overall trends. In terms of the number of patients with specific mental disorders, the number of patients with schizophrenia decreased very slightly, whereas the number of patients with other mental disorders increased during the pandemic. For each disorder, the number of hospital admissions decreased, while the number of outpatient visits increased (Table 1).

### Trend analysis of quarterly mental health service use patterns according to the principal diagnosis before and during the COVID-19 pandemic

After the outbreak of COVID-19, the overall number of admissions and length of hospitalization decreased significantly for admissions through the ED and elective admissions, excluding the number of admission of patients with bipolar disorder, anxiety disorder, and sleep disorder. In addition, the number of outpatient visits of patients with dementia, schizophrenia, and bipolar disorder was significantly reduced. However, the number of outpatient visits in patients with depression, anxiety disorder, and sleep disorders decreased significantly after the outbreak of COVID-19. The number of emergency care visits decreased significantly after the outbreak of COVID-19 for all mental disorders (Supplementary Material 1).

**Figure 1.**
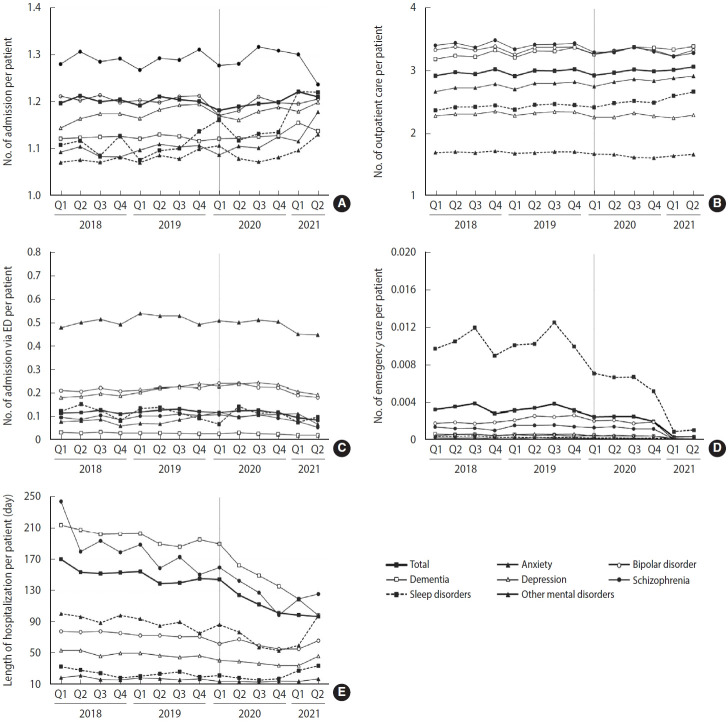
Quarterly trends of mental health service use patterns according to the principal diagnosis. Each graph represents the trend of indicator: (A) number of admission per patient, (B) number of outpatient care per patient, (C) number of admission via emergency department (ED) per patient, (D) number of emergency care per patient, and (E) length of hospitalization per patient. Vertical lines shown in Q1 2020 represent when the coronavirus disease 2019 pandemic first started.

Trend analysis was performed on mental health service utilization for major mental disorders by each quarter between January 2018 and June 2021, representing the period before and during the COVID-19 pandemic (Figure 1). For all mental disorders, the number of hospital admissions per patient increased during Q1 of 2021, whereas ED visits and the length of hospital stay tended to decrease during Q1 and Q2 of 2021. Specifically, for schizophrenia, hospital admissions and outpatient visits tended to decrease in 2021 when the pandemic became more severe; in particular, admissions via the ED and ED visits decreased, although only slightly. For bipolar disorder, the number of hospital admissions and outpatient visits decreased in the early stage of the pandemic, but subsequently recovered to a level similar to the previous year. Furthermore, the number of outpatient visits decreased slightly in 2021; admissions via the ED also decreased from the beginning of 2021, and ED visits decreased substantially in 2021.

For depression, the number of hospital admissions showed an increasing pattern in the early stage of the pandemic. The number of outpatient visits decreased slightly in Q2 of 2020 but showed an almost flat trend during the study period. While there was no significant difference in admissions via the ED, ED visits tended to decrease in 2021. The length of hospital stay decreased in 2020 but showed a pattern of recovery in 2021. For anxiety disorder, the number of hospital admissions increased during Q4 of 2020 with the prolongation of the pandemic and continued to increase throughout 2021. Admissions via the ED also showed an increasing trend in 2020 and 2021, compared with before the pandemic. The number of outpatient visits showed a slightly decreasing trend in 2021, while the number of ED visits decreased substantially. For sleep disorders, the number of hospital admissions increased during the early stage of the pandemic as well as in Q1 and Q2 of 2021, while admissions via the ED decreased in 2021. The number of outpatient visits increased during Q1 and Q2 of 2021, while ED visits decreased in 2021. There was no change in the length of hospital stay except in Q2 of 2021.

### Comparison of mental health service use patterns before and during the COVID-19 pandemic

Interrupted time series analysis was performed to identify the impact of the first wave of COVID-19 on mental health service utilization. The results showed an overall decreasing trend in the number of hospital admissions; the number of hospital admissions per patient decreased significantly by 1.2% and 0.7% at the outbreak of the pandemic (Q1 of 2020) and thereafter, respectively. The length of hospital stay also showed an overall decreasing trend. However, the length of hospital stay for all hospital admissions (including admissions via the ED) increased by 1.8% at the outbreak of the pandemic, but decreased by 20.2% thereafter. The number of admission via the ED decreased by 3.3% after the outbreak of COVID-19. The number of outpatient visits showed an overall increasing trend, but the number of outpatient visits per patient showed a significant decrease by 3.0% and 2.2% immediately post-outbreak of the pandemic and thereafter, respectively. The number of ED visits for mental health per patient also decreased by 22.4% and 22.4% at the outbreak of the pandemic and thereafter, respectively (Table 2).

### Comparison of mental health service use patterns before and during the COVID-19 pandemic according to principal diagnosis

**Figure 2.**
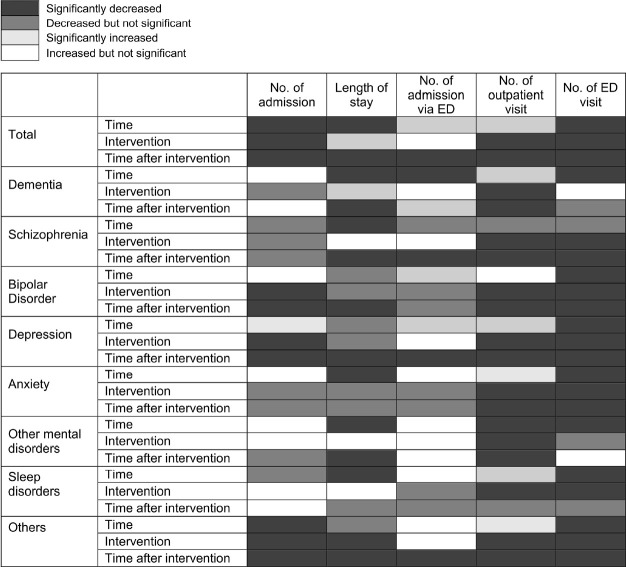
Changes in mental health service use after the coronavirus disease 2019 (COVID-19) outbreak according to the principal diagnosis, using interrupted time series analysis. Time represents the time since the start of the study period, “intervention” indicates whether the time is before (0) or after (1) the occurrence of COVID-19, and time after intervention represents the time elapsed after the occurrence of COVID-19, taking a value of 0 prior to COVID-19. ED, emergency department.

As shown in Figure 2, inpatient and outpatient use decreased after the outbreak of COVID-19. An interrupted time series analysis by major mental disorders was performed to analyze the differences in mental health service utilization before and during the pandemic (Figure 2, Supplementary Material 2). At the time of COVID-19, mental health service use decreased or increased depending on the disease, but after COVID-19, medical use decreased for most mental disorders (excluding other mental disorders and sleep disorders). The number of hospital admissions per patient showed significant decreases for bipolar disorder, depression, and others at the time of the pandemic and thereafter. The number of hospital admissions per patient decreased by a large margin for bipolar disorder (3.3%) at the COVID-19 outbreak and for depression (3.0%) during the pandemic.

Regarding the length of hospital stay, at the time of the pandemic, there was a significant increase for dementia (3.9%) and a significant decrease for others (7.6%). Other mental disorders, including schizophrenia, bipolar disorder, depression, and anxiety disorder, showed no significant decrease in length of hospital stay. After the outbreak of the pandemic, the length of hospital stay for patients with dementia, schizophrenia, bipolar disorder, depression, other mental disorders, and others showed a significant decrease of 25.5%, 9.8%, 9.5%, 13.5%, 16.4%, and 14.9%, respectively; however, there were no significant changes for patients with anxiety and sleep disorder. Furthermore, the number of admissions via the ED showed no significant change at the time of the pandemic. However, after the COVID-19 pandemic, there was a significant decrease in admission via the ED for schizophrenia, depression, and others, with depression showing the largest decrease (5.4%).

There was a significant decrease in the number of outpatient visits immediately after the outbreak of the pandemic and thereafter for all mental disorders except sleep disorders, which showed a significant decrease at the time of the pandemic only. In particular, bipolar disorder and schizophrenia showed decreases of 3.5% and 3.4%, respectively, at the time of the pandemic, which were the largest decreases among all mental disorders, except for the category of others. During the pandemic, schizophrenia and dementia showed decreases of 3.2% and 3.6%, respectively, which were the largest decreases in the number of outpatient visits among all mental disorders.

There was an overall decrease in the number of admissions via the ED for all mental disorders. All mental disorders, except dementia and other mental disorders, showed a significant decrease at the time of the pandemic and thereafter. During the pandemic, schizophrenia (19.2%), bipolar disorder (22.3%), anxiety (3.4%), depression (17.4%), and others (40.9%) showed significant decreases in the number of admissions via the ED.

## DISCUSSION

This study used nationwide health insurance claims data to examine the impact of the pandemic on health service utilization for major mental disorders, including hospital admissions, outpatient visits, and ED visits. With limited access to traditional healthcare services during the pandemic, the use of telemedicine received substantial attention [13]; in Korea, telephone consultations and prescriptions via telemedicine were temporarily permitted during the pandemic [14]. This study also included data for tele-mental health service utilization.

The number of patients diagnosed with a mental disorder increased after the outbreak of the pandemic, with a decrease in inpatients and an increase in outpatients. Patients aged 20-44 years and above 65 years showed increases in their utilization of outpatient services. In addition, the number of female outpatients increased more than that of male outpatients. This was presumably because young women used mental health services to address depression. In 2 cohort studies from England that investigated mental health during the pandemic, the prevalence of anxiety was higher among younger patients, female patients, those with existing psychiatric/physical problems, and those with socioeconomic difficulties [15]. In Japan, young women aged < 40 years showed the highest increase in suicide rates after the outbreak of the pandemic, indicating that this population faced deteriorating mental health and significant economic impacts due to the pandemic [16]. In a Korean study on depression among the general population during the pandemic, young age, along with having a mental disorder and being a smoker, were identified as risk factors [17]. Thus, young females may have experienced a significant impact on mental health due to COVID-19, and their mental health service utilization may have also increased. Therefore, priority should be given to this group with respect to mental health countermeasures.

As patients with mental disorders have been at an increased risk of infection and vulnerable to complications since the beginning of the pandemic, research has claimed that it is necessary to accelerate the community management of mental disorders and reduce admissions to overcrowded psychiatric hospitals as much as possible [18]. In fact, mass infections in psychiatric hospitals led to rapid discharge to prevent the spread of infection, and the use of hospitals was avoided due to concerns regarding infection [19]. Thus, while the number of inpatients decreased for these reasons, healthcare needs increased due to COVID-19, leading to an increasing trend in the number of outpatients.

For severe mental disorders, such as schizophrenia and bipolar disorder, continued use of outpatient services is important for preventing recurrence and ensuring favorable progress [20]. The current findings showed a slight increase in the number of outpatients, but a decrease in the number of outpatient visits during the pandemic. At the time of the pandemic, schizophrenia and bipolar disorder showed the largest decreases in outpatient visits, whereas the magnitude of the decrease in outpatient visits for schizophrenia was larger than that of other mental disorders during the pandemic. Another study in Korea predicted that outpatient visits and hospital admissions for patients with schizophrenia would decrease by 3.6% during 2020 (i.e., the early stage of the pandemic) [21]. In a study that analyzed the utilization of inpatient mental healthcare in Rhineland, Germany, during the first wave of the pandemic, it was found that hospitalization rates decreased by 25% relative to the previous year, with a decrease of 34% for affective disorders and 10% for schizophrenia, which were smaller proportional decreases than for other disorders [7].

In Korea, inpatient care plays a central role in the treatment of schizophrenia; it is the only advanced country with longer average stays in the psychiatric ward and has a higher number of beds than other countries [22,23]. Although the number of hospital admissions for schizophrenia showed no significant change in the regression analysis, a decreasing trend was found as the pandemic worsened, and the length of hospital stay also decreased. This may have been because patients under long-term hospitalization were discharged due to COVID-19, and the admission procedure was more difficult due to the mandatory requirement of a negative COVID-19 test result on admission.

Compared with before the pandemic, the decrease in overall mental health service utilization was due to a decrease in the length of hospital stay, admissions via the ED, and ED visits. In particular, a decrease in ED visits starting in the third wave of COVID-19 in Korea was prominent for all disorders, and ED visits did not recover to the previous level. A Swiss study that investigated changes in psychiatric emergency admissions between 8 weeks before and after a COVID-19 lockdown found increases in suicidal behavior, psychomotor agitation, and involuntary admissions after the lockdown [24]. In an Italian observational study on psychiatric emergencies over 6 months during the pandemic, there was a statistically significant decrease in psychiatric consultation in the ED and admissions to the psychiatric ward [25]. In another study from Italy, psychiatric consultations decreased by 37.5% during a lockdown, and a decline of 17.9% persisted even after the lockdown; however, the number of involuntary admissions increased compared to 2019 [26]. ED visits may decrease during the pandemic, but once the COVID-19 situation improves, ED visits may increase in patients with serious psychiatric problems, which indicates the need to prepare for psychiatric emergency healthcare during the pandemic.

In Korea, compared to before the pandemic, the number of outpatient visits decreased at the outbreak of the pandemic and thereafter. However, the overall number of outpatient visits increased significantly during the pandemic. A Korean study that investigated mental health service use among psychiatric outpatients in a tertiary hospital for 3 months during the early stage of the pandemic reported that outpatient visits decreased sharply during the pandemic, while anxiety disorder and depression decreased significantly, consistent with our findings [27]. In the United States, outpatient mental health service use decreased by half during several months in the early stage of the pandemic, and subsequently, telehealth accounted for 47.9% of the outpatient services used, leading to a rapid recovery of the declining rate [28]. Korea showed a relatively good response to COVID-19 and maintained the continued use of healthcare services, without any shutdown as in other countries [21]. The present study indicates that the worsening of mental health indicators due to COVID-19 was responsible for an increase in mental health needs, which led to an increase in the overall utilization of outpatient services. These findings demonstrate the need for preparation at the national level to handle increased mental health needs when COVID-19 becomes endemic.

For dementia and schizophrenia, which have high disease severity and are likely to require hospitalization, the number of hospital admissions showed no significant difference in the time series analysis, while the length of hospital stay and number of outpatient visits showed a decrease during the pandemic. The decrease in the number of outpatient visits for dementia, schizophrenia, and bipolar disorder, and the decrease in the number of hospital admissions for bipolar disorder may be attributed more to factors such as not providing the necessary care rather than the effect of the pandemic on the onset of the disorder (Figure 2). A United States cohort study reviewed the electronic medical records of 61.9 million Americans and reported that patients with dementia had a higher risk of COVID-19 infection and hospitalization than adults (aged ≥ 18 years) and older adult patients [29]; meanwhile, a meta-analysis reported that patients with mood disorders had higher risks of hospitalization and death due to COVID-19 [30]. However, the present study is significant because it is the first to identify health service utilization by all patients with dementia.

Although the number of patients with depression, anxiety, and sleep disorders increased after the COVID-19 outbreak, the number of outpatient visits during the pandemic showed a decrease in the interrupted time series analysis (Supplementary Material 2). A national health survey in Korea reported that depression, anxiety, insomnia, and suicidal ideation increased during the COVID19 pandemic [3]. A follow-up questionnaire survey on 3,000 adults in the United Kingdom reported an increase in suicidal ideation, a decrease in anxiety, and no significant change in depression over time [2]. In a meta-analysis of the scale of sleep problems during the pandemic, the estimated prevalence of sleep problems was 18% among the general population, 57% among COVID-19 patients, and 31% among healthcare professionals; the results also indicated an association between sleep problems and depression and anxiety levels [31]. While it is opined that mental health survey results are not directly correlated with increased health service utilization and caution is needed in interpreting the results, this study contributed by showing that anxiety, depression, and sleep disorders increased during the pandemic, but the number of outpatient visits per patient decreased.

The present study has the following limitations. First, patients with mental disorders were selected based on the principal diagnoses in medical claims data. However, because such data are for payment of medical fees, inaccurate diagnostic codes may be included in some cases. Second, data from the second half of 2021 were not included, as nationwide data for that period had not been processed at the time of the study. Consequently, changes in health service utilization during the process of recovery from the third wave of COVID-19 were not accounted for in the study. Third, the data used in the present study included tele-mental health service utilization, but detailed analyses by disorder, age, and sex were not performed. Additional analyses may be needed to investigate the significance of tele-mental health services due to the pandemic. Fourth, the decrease in the number of inpatient visits and the increase in the number of outpatient visits during the COVID-19 pandemic identified in this study may follow the adjustment of existing psychiatric beds to allocate beds for the medical care of the COVID-19 hospitalization. This possibility should be considered in analysis and interpretation. Fifth, in the COVID-19 pandemic, the problem of substance use disorders such as alcohol and drugs is of considerable significance, but it was not included in the analysis of this study. Sixth, results were derived based on the care provided and do not precisely account for the actual care needed. Follow-up studies are needed on various aspects of health outcomes based on health service utilization to understand the extent of care requirements.

In conclusion, the findings showed that the overall use of mental health service utilization increased during the pandemic, whereas health service utilization decreased, especially via the ED. As the pandemic worsened, intense social distancing policies were implemented, resulting in restrictions on outpatient visits. There was also a tendency not to receive treatment due to fear of infection during the treatment process [15]. As a result, outpatient visits for severe mental illnesses (schizophrenia and bipolar disorder) significantly decreased; therefore, countermeasures are needed to maintain the continuity of care. It is necessary to establish mental healthcare policies for COVID-19 based on these findings.

## Figures and Tables

**Figure f3-epih-45-e2023022:**
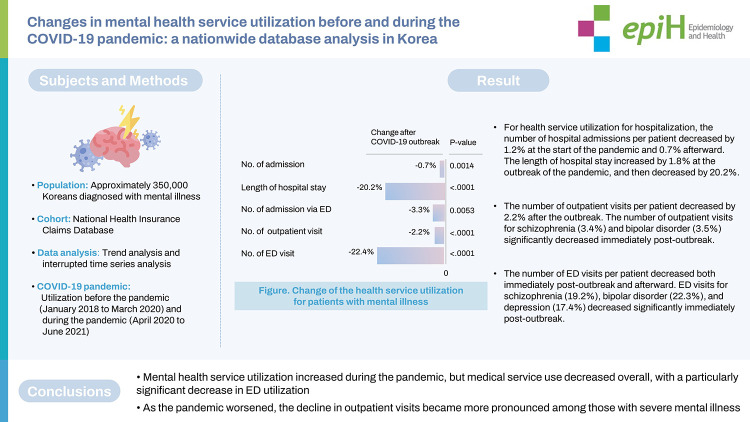


**Table 1.** Characteristics of patients with mental disorders from January 2018 to June 2021

Characteristics		All					Inpatient treatment					Outpatient treatment				
		2018	2019	2020	Until June 2021	p-value^1^	2018	2019	2020	Until June 2021	p-value^1^	2018	2019	2020	Until June 2021	p-value^1^
Total (n)		3,319,429	3,537,643	3,636,506	3,009,609		239,099	232,858	196,911	126,831		3,216,342	3,438,835	3,551,093	2,939,460	
Age (yr)						<0.001					<0.001					<0.001
	Mean±SD	56.1±21.7	56.0±22.0	55.9±22.2	56.0±22.3	<0.001	64.7±21.3	64.9±21.6	65.3±21.8	63.5±21.4	<0.001	55.5±21.6	55.5±21.9	55.4±22.1	55.7±22.3	<0.001
	<20	219,106 (6.6)	236,870 (6.7)	232,054 (6.4)	194,433 (6.5)		6,845 (2.9)	6,934 (3.0)	5,624 (2.9)	4,031 (3.2)		218,027 (6.8)	235,798 (6.9)	231,304 (6.5)	193,848 (6.6)	
	20-44	750,740 (22.6)	822,097 (23.2)	885,838 (24.4)	731,699 (24.3)		37,525 (15.7)	36,899 (15.9)	31,560 (16.0)	20,465 (16.1)		743,427 (23.1)	815,334 (23.7)	880,312 (24.8)	725,447 (24.7)	
	45-64	1,027,560 (31.0)	1,060,409 (30.0)	1,051,856 (28.9)	855,208 (28.4)		64,843 (27.1)	61,391 (26.4)	48,947 (24.9)	37,882 (29.9)		1,005,387 (31.3)	1,039,921 (30.2)	1,035,302 (29.2)	834,225 (28.4)	
	65-79	816,763 (24.6)	854,081 (24.1)	871,952 (24.0)	722,667 (24.0)		48,732 (20.4)	45,931 (19.7)	38,611 (19.6)	24,664 (19.5)		793,319 (24.7)	832,376 (24.2)	853,314 (24.0)	707,752 (24.1)	
	≥80	505,260 (15.2)	564,186 (16.0)	594,806 (16.4)	505,602 (16.8)		81,154 (33.9)	81,703 (35.1)	72,169 (36.7)	39,789 (31.4)		456,182 (14.2)	515,406 (15.0)	550,861 (15.5)	478,188 (16.3)	
Sex						<0.001					0.010					<0.001
	Male	1,320,398 (39.8)	1,404,810 (39.7)	1,432,104 (39.4)	1,174,478 (39.0)		106,036 (44.4)	102,202 (43.9)	86,370 (43.9)	59,669 (47.1)		1,276,870 (39.7)	1,363,694 (39.7)	1,396,243 (39.3)	1,140,550 (38.8)	
	Female	1,999,031 (60.2)	2,132,833 (60.3)	2,204,402 (60.6)	1,835,131 (61.0)		133,063 (55.7)	130,656 (56.1)	110,541 (56.1)	67,162 (53.0)		1,939,472 (60.3)	2,075,141 (60.3)	2,154,850 (60.7)	1,798,910 (61.2)	
Health insurance type						<0.001					0.002					<0.001
	National Health Insurance	2,933,757 (88.4)	3,133,378 (88.6)	3,224,818 (88.7)	2,640,264 (87.7)		173,756 (72.7)	169,751 (72.9)	143,806 (73.0)	83,862 (66.1)		2,857,444 (88.8)	3,059,907 (89.0)	3,162,006 (89.0)	2,597,046 (88.4)	
	Medical Aid	385,672 (11.6)	404,265 (11.4)	411,688 (11.3)	369,345 (12.3)		65,343 (27.3)	63,107 (27.1)	53,105 (27.0)	42,969 (33.9)		358,898 (11.2)	378,928 (11.0)	389,087 (11.0)	342,414 (11.7)	
Principal diagnosis						<0.001					<0.001					<0.001
	Dementia	507,391 (15.3)	551,919 (15.6)	566,694 (15.6)	484,983 (16.1)		108,328 (45.3)	107,723 (46.3)	94,846 (48.2)	53,219 (42.0)		440,870 (13.7)	486,470 (14.2)	508,026 (14.3)	447,910 (15.2)	
	Schizophrenia	193,933 (5.8)	195,250 (5.5)	195,150 (5.4)	192,515 (6.4)		36,406 (15.2)	34,091 (14.6)	28,900 (14.7)	27,424 (21.6)		184,771 (5.7)	187,026 (5.4)	187,667 (5.3)	176,866 (6.0)	
	Bipolar disorder	89,082 (2.7)	97,228 (2.8)	103,465 (2.9)	97,899 (3.3)		11,201 (4.7)	11,123 (4.8)	9,590 (4.9)	6,200 (4.9)		88,052 (2.7)	96,251 (2.8)	102,634 (2.9)	96,639 (3.3)	
	Depression	777,246 (23.4)	837,020 (23.7)	888,230 (24.4)	752,269 (25.0)		21,484 (9.0)	21,577 (9.3)	17,829 (9.1)	10,191 (8.0)		774,702 (24.1)	834,390 (24.3)	886,074 (25.0)	750,362 (25.5)	
	Anxiety disorder	614,631 (18.5)	639,349 (18.1)	663,209 (18.2)	533,760 (17.7)		8,486 (3.6)	7,999 (3.4)	6,473 (3.3)	3,570 (2.8)		612,714 (19.1)	637,544 (18.5)	661,793 (18.6)	532,877 (18.1)	
	Other mental disorders^2^	220,710 (6.7)	257,199 (7.3)	260,685 (7.2)	200,412 (6.7)		5,099 (2.1)	4,277 (1.8)	2,859 (1.5)	1,786 (1.4)		218,325 (6.8)	255,477 (7.4)	259,798 (7.3)	199,538 (6.8)	
	Sleep disorders	299,944 (9.0)	313,363 (8.9)	323,005 (8.9)	246,773 (8.2)		2,527 (1.1)	2,461 (1.1)	1,923 (1.0)	864 (0.7)		299,582 (9.3)	312,992 (9.1)	322,651 (9.1)	246,555 (8.4)	
	Others	616,492 (18.6)	646,315 (18.3)	636,068 (17.5)	500,998 (16.7)		45,568 (19.1)	43,607 (18.7)	34,491 (17.5)	23,577 (18.6)		597,326 (18.6)	628,685 (18.3)	622,450 (17.5)	488,713 (16.6)	

Values are presented as frequency (%). SD, standard deviation.

1 p-values for association years (2018 to 2020) and characteristics based on analysis of variance or chi-square test.

2 Other mental disorders: other mental disorders due to brain damage and dysfunction and to physical disease, others: remaining mental disorders.

**Table 2.** Changes in mental health service use after the COVID-19 outbreak using interrupted time series analysis

Variables		No. of admissions			Length of hospitalization			No. of admissions via the ED			No. of outpatient visits			No. of ED visit		
		Est.	SE	p-value	Est.	SE	p-value	Est.	SE	p-value	Est.	SE	p-value	Est.	SE	p-value
Age (yr)																
	<20	Reference			Reference			Reference			Reference			Reference		
	20-44	-0.1250	0.0040	<0.001	11.8108	1.3295	<0.001	-0.0808	0.0022	<0.001	-0.2462	0.0020	<0.001	0.0046	0.0001	<0.001
	45-64	-0.2257	0.0039	<0.001	45.6698	1.3007	<0.001	-0.1699	0.0021	<0.001	-0.7379	0.0020	<0.001	0.0036	0.0001	<0.001
	65-79	-0.2722	0.0041	<0.001	45.5034	1.3751	<0.001	-0.1821	0.0022	<0.001	-0.9320	0.0021	<0.001	0.0020	0.0001	<0.001
	≥80	-0.2710	0.0042	<0.001	62.8048	1.4272	<0.001	-0.2058	0.0023	<0.001	-0.9174	0.0023	<0.001	0.0022	0.0001	<0.001
Health insurance type																
	National Health Insurance	Reference			Reference			Reference			Reference			Reference		
	Medical Aid	-0.0318	0.0015	<0.001	53.5860	0.4986	<0.001	-0.0867	0.0008	<0.001	0.5940	0.0013	<0.001	0.0013	0.0000	<0.001
Sex																
	Male	Reference			Reference			Reference			Reference			Reference		
	Female	-0.0560	0.0014	<0.001	6.6630	0.4596	<0.001	0.0477	0.0007	<0.001	0.0801	0.0009	<0.001	0.0004	0.0000	<0.001
Principal diagnosis																
	Dementia	Reference			Reference			Reference			Reference			Reference		
	Schizophrenia	0.1021	0.0026	<0.001	-2.4377	0.8715	0.005	0.0461	0.0014	<0.001	0.6306	0.0021	<0.001	0.0000	0.0001	0.749
	Bipolar disorder	0.0403	0.0037	<0.001	-75.0492	1.2381	<0.001	0.1437	0.0020	<0.001	0.8485	0.0027	<0.001	0.0018	0.0001	<0.001
	Depression	0.0036	0.0028	0.196	-100.6518	0.9461	<0.001	0.1644	0.0015	<0.001	0.8235	0.0016	<0.001	-0.0001	0.0000	0.007
	Anxiety disorder	-0.0464	0.0037	<0.001	-117.3339	1.2421	<0.001	0.3240	0.0020	<0.001	0.3577	0.0017	<0.001	0.0042	0.0000	<0.001
	Other mental disorders^1^	-0.0366	0.0049	<0.001	-74.9949	1.6445	<0.001	0.0496	0.0027	<0.001	-0.5706	0.0021	<0.001	-0.0006	0.0001	<0.001
	Sleep disorders	-0.0290	0.0062	<0.001	-107.6685	2.0674	<0.001	0.1546	0.0033	<0.001	0.1398	0.0020	<0.001	-0.0008	0.0001	<0.001
	Others	0.0210	0.0024	<0.001	-71.4373	0.8226	<0.001	0.1614	0.0013	<0.001	0.0298	0.0019	<0.001	0.0093	0.0001	<0.001
Time^2^		-0.0007	0.0003	0.022	-1.8182	0.1066	<0.001	0.0004	0.0002	0.031	0.0086	0.0002	<0.001	-0.0001	0.0000	<0.001
Intervention		-0.0138	0.0030	<0.001	2.2382	0.9989	0.025	0.0021	0.0016	0.188	-0.0882	0.0019	<0.001	-0.0008	0.0001	<0.001
Time after intervention		-0.0089	0.0028	0.001	-25.4672	0.9347	<0.001	-0.0042	0.0015	0.005	-0.0659	0.0019	<0.001	-0.0008	0.0001	<0.001

COVID, coronavirus disease 2019 ; ED, emergency department; Est, estimate; SE, standard error.

1 Other mental disorders: other mental disorders due to brain damage and dysfunction and to physical disease, others: remaining mental disorders.

2 Time represents the time since the start of the study period, “intervention” indicates whether the time is before (0) or after (1) the occurrence of COVID-19, and time after intervention represents the time elapsed after the occurrence of COVID-19, taking a value of 0 prior to COVID-19.

## SUPPLEMENTARY MATERIALS

Supplementary Material 1. Change of Mental Health Use Before and After COVID-19 pandemic According to Principal Diagnosis

Supplementary Material 2. Change of Mental Health Use After COVID-19 pandemic According to Principal Diagnosis Using Interrupted Time Series Analysis
