# Supplementary material for: Changes in mental health service utilization before and during the COVID-19 pandemic: a nationwide database analysis in Korea
Source: Epidemiol Health. 2023 Feb 14;45:e2023022. doi: 10.4178/epih.e2023022 (PMC10266929; doi:10.4178/epih.e2023022)
Supplement: Supplementary Material 1. — Change of Mental Health Use Before and After COVID-19 pandemic According to Principal Diagnosis [file epih-45-e2023022-Supplementary-1.docx]

**Supplementary Material 1. Change of Mental Health Use Before and After COVID-19 pandemic According to Principal Diagnosis**

|  |  | January 2018 ~ February 2020 | March 2020 ~ June 2021 | P-values |
| --- | --- | --- | --- | --- |
| Number of admission | All | 1.20±0.64 | 1.19±0.65 | <.0001 |
|  | Dementia | 1.13±0.40 | 1.13±0.42 | 0.0328 |
|  | Schizophrenia | 1.32±0.91 | 1.28±0.88 | <.0001 |
|  | Bipolar Disorder | 1.27±0.77 | 1.26±0.77 | 0.1330 |
|  | Depression | 1.23±0.72 | 1.22±0.72 | 0.0219 |
|  | Anxiety disorder | 1.17±0.57 | 1.17±0.62 | 0.2244 |
|  | Other mental disorders | 1.13±0.48 | 1.12±0.61 | 0.1758 |
|  | Sleep disorder | 1.16±0.52 | 1.16±0.47 | 0.7421 |
|  | Others | 1.25±0.74 | 1.22±0.75 | <.0001 |
| Length of hospitalization | All | 132.20±230.85 | 111.31±136.43 | <.0001 |
|  | Dementia | 174.29±262.79 | 138.25±154.04 | <.0001 |
|  | Schizophrenia | 162.86±261.34 | 141.36±133.90 | <.0001 |
|  | Bipolar Disorder | 74.70±147.14 | 71.59±96.79 | 0.0062 |
|  | Depression | 52.52±125.20 | 46.47±81.39 | <.0001 |
|  | Anxiety disorder | 34.24±106.95 | 29.41±66.93 | <.0001 |
|  | Other mental disorders | 96.41±191.63 | 81.31±122.09 | <.0001 |
|  | Sleep disorder | 53.44±136.19 | 47.22±90.84 | 0.0031 |
|  | Others | 89.00±170.91 | 83.95±109.04 | <.0001 |
| Number of admission via emergency department | All | 0.127±0.362 | 0.125±0.360 | 0.0005 |
|  | Dementia | 0.033±0.186 | 0.032±0.186 | 0.2741 |
|  | Schizophrenia | 0.104±0.339 | 0.086±0.308 | <.0001 |
|  | Bipolar Disorder | 0.235±0.487 | 0.240±0.503 | 0.3058 |
|  | Depression | 0.256±0.492 | 0.274±0.511 | <.0001 |
|  | Anxiety disorder | 0.416±0.551 | 0.421±0.561 | 0.3749 |
|  | Other mental disorders | 0.089±0.294 | 0.102±0.312 | 0.0077 |
|  | Sleep disorder | 0.212±0.435 | 0.217±0.438 | 0.632 |
|  | Others | 0.218±0.444 | 0.210±0.437 | 0.0002 |
| Number of outpatient visit | All | 2.97±2.36 | 2.97±2.34 | <.0001 |
|  | Dementia | 2.39±1.73 | 2.35±1.71 | <.0001 |
|  | Schizophrenia | 3.48±2.50 | 3.40±2.43 | <.0001 |
|  | Bipolar Disorder | 3.61±2.62 | 3.58±2.62 | <.0001 |
|  | Depression | 3.48±2.54 | 3.53±2.55 | <.0001 |
|  | Anxiety disorder | 2.97±2.39 | 3.03±2.39 | <.0001 |
|  | Other mental disorders | 1.83±1.46 | 1.77±1.39 | <.0001 |
|  | Sleep disorder | 2.67±2.14 | 2.72±2.14 | <.0001 |
|  | Others | 2.87±2.48 | 2.88±2.43 | <.0001 |
| Number of emergency care visit | All | 0.0040±0.0720 | 0.0027±0.0596 | <.0001 |
|  | Dementia | 0.0009±0.0342 | 0.0007±0.0299 | <.0001 |
|  | Schizophrenia | 0.0028±0.0608 | 0.0022±0.0582 | <.0001 |
|  | Bipolar Disorder | 0.0043±0.0752 | 0.0034±0.0658 | <.0001 |
|  | Depression | 0.0019±0.0509 | 0.0014±0.0430 | <.0001 |
|  | Anxiety disorder | 0.0064±0.0946 | 0.0049±0.0836 | <.0001 |
|  | Other mental disorders | 0.0004±0.0210 | 0.0003±0.0174 | 0.0002 |
|  | Sleep disorder | 0.0010±0.0337 | 0.0007±0.0299 | <.0001 |
|  | Others | 0.0112±0.1169 | 0.0068±0.0883 | <.0001 |

Tables were expressed by Mean±standard deviation

P-values from student’s t-test

Other mental disorders: Other mental disorders due to brain damage and dysfunction and to physical disease, others: remainder of mental disorders
