# Supplementary material for: Changes in mental health service utilization before and during the COVID-19 pandemic: a nationwide database analysis in Korea
Source: Epidemiol Health. 2023 Feb 14;45:e2023022. doi: 10.4178/epih.e2023022 (PMC10266929; doi:10.4178/epih.e2023022)
Supplement: Supplementary Material 2. — Change of Mental Health Use After COVID-19 pandemic According to Principal Diagnosis Using Interrupted Time Series Analysis [file epih-45-e2023022-Supplementary-2.docx]

**Supplementary Material 2. Change of Mental Health Use After COVID-19 pandemic According to Principal Diagnosis Using Interrupted Time Series Analysis**

| Principal diagnosis |  | Number of admission | | | Length of stay | | | Number of admission via  Emergency department | | | Number of outpatient visit | | | Number of Emergency department visit | | |
| --- | --- | --- | --- | --- | --- | --- | --- | --- | --- | --- | --- | --- | --- | --- | --- | --- |
|  |  | Est | SE | P | Est. | SE | P | Est. | SE | P | Est. | SE | P | Est. | SE | P |
| Dementia | Age |  |  |  |  |  |  |  |  |  |  |  |  |  |  |  |
|  | <20y | Ref. |  |  | Ref. |  |  | Ref. |  |  | Ref. |  |  | Ref. |  |  |
|  | 20-44y | 0.2706 | 0.1799 | 0.1325 | 98.0033 | 111.112 | 0.3778 | 0.0530 | 0.0850 | 0.533 | -3.5997 | 0.1863 | <.0001 | 0.0006 | 0.0036 | 0.8715 |
|  | 45-64y | 0.1476 | 0.1788 | 0.409 | 150.618 | 110.440 | 0.1726 | 0.0396 | 0.0844 | 0.6395 | -3.2794 | 0.1837 | <.0001 | 0.0014 | 0.0035 | 0.6917 |
|  | 65-79y | 0.1464 | 0.1787 | 0.4129 | 111.654 | 110.427 | 0.312 | 0.0429 | 0.0844 | 0.6117 | -3.3177 | 0.1837 | <.0001 | 0.0008 | 0.0035 | 0.8136 |
|  | ≥80y | 0.1383 | 0.1787 | 0.439 | 123.781 | 110.426 | 0.2623 | 0.0318 | 0.0844 | 0.7062 | -3.2234 | 0.1837 | <.0001 | 0.0010 | 0.0035 | 0.7784 |
|  | Health Insurance type |  |  |  |  |  |  |  |  |  |  |  |  |  |  |  |
|  | Health Insurance | Ref. |  |  | Ref. |  |  | Ref. |  |  | Ref. |  |  | Ref. |  |  |
|  | Medical aid | -0.0193 | 0.0016 | <.0001 | 21.5595 | 0.9637 | <.0001 | -0.0140 | 0.0007 | <.0001 | 0.4096 | 0.0023 | <.0001 | 0.0003 | 0.00004 | <.0001 |
|  | Sex |  |  |  |  |  |  |  |  |  |  |  |  |  |  |  |
|  | Male | Ref. |  |  | Ref. |  |  | Ref. |  |  | Ref. |  |  | Ref. |  |  |
|  | Female | -0.0432 | 0.0014 | <.0001 | 45.4073 | 0.8393 | <.0001 | -0.0057 | 0.0006 | <.0001 | 0.0850 | 0.0018 | <.0001 | -0.0003 | 0.00003 | <.0001 |
|  | Time | 0.0001 | 0.0003 | 0.7744 | -2.4344 | 0.1895 | <.0001 | -0.0008 | 0.0001 | <.0001 | 0.0026 | 0.0004 | <.0001 | -0.00004 | 0.00001 | <.0001 |
|  | Intervention | -0.0044 | 0.0028 | 0.1114 | 6.3844 | 1.7011 | 0.0002 | 0.0010 | 0.0013 | 0.4642 | -0.0838 | 0.0037 | <.0001 | 0.00002 | 0.0001 | 0.8080 |
|  | Time after intervention | 0.0037 | 0.0027 | 0.1680 | -41.8040 | 1.6452 | <.0001 | 0.0038 | 0.0013 | 0.0022 | -0.0850 | 0.0035 | <.0001 | -0.00001 | 0.0001 | 0.8667 |
| Schizophrenia | Age |  |  |  |  |  |  |  |  |  |  |  |  |  |  |  |
|  | <20y | Ref. |  |  | Ref. |  |  | Ref. |  |  | Ref. |  |  | Ref. |  |  |
|  | 20-44y | -0.0267 | 0.0179 | 0.1363 | 44.8698 | 4.7401 | <.0001 | -0.0954 | 0.0066 | <.0001 | -0.7863 | 0.0146 | <.0001 | -0.0050 | 0.0004 | <.0001 |
|  | 45-64y | -0.1822 | 0.0179 | <.0001 | 103.163 | 4.7422 | <.0001 | -0.1410 | 0.0066 | <.0001 | -1.0547 | 0.0146 | <.0001 | -0.0073 | 0.0004 | <.0001 |
|  | 65-79y | -0.2620 | 0.0188 | <.0001 | 116.941 | 4.9644 | <.0001 | -0.1562 | 0.0069 | <.0001 | -1.2358 | 0.0152 | <.0001 | -0.0081 | 0.0004 | <.0001 |
|  | ≥80y | -0.3497 | 0.0256 | <.0001 | 103.728 | 6.7659 | <.0001 | -0.1681 | 0.0095 | <.0001 | -1.2331 | 0.0184 | <.0001 | -0.0084 | 0.0005 | <.0001 |
|  | Health Insurance type |  |  |  |  |  |  |  |  |  |  |  |  |  |  |  |
|  | Health Insurance | Ref. |  |  | Ref. |  |  | Ref. |  |  | Ref. |  |  | Ref. |  |  |
|  | Medical aid | -0.2222 | 0.0047 | <.0001 | 72.4166 | 1.2336 | <.0001 | -0.0895 | 0.0017 | <.0001 | 0.5755 | 0.0035 | <.0001 | -0.0002 | 0.0001 | 0.006 |
|  | Sex |  |  |  |  |  |  |  |  |  |  |  |  |  |  |  |
|  | Male | Ref. |  |  | Ref. |  |  | Ref. |  |  | Ref. |  |  | Ref. |  |  |
|  | Female | -0.0613 | 0.0044 | <.0001 | -17.0128 | 1.1594 | <.0001 | 0.0201 | 0.0016 | <.0001 | 0.0175 | 0.0033 | <.0001 | 0.0006 | 0.0001 | <.0001 |
|  | Time | -0.0021 | 0.0011 | 0.0619 | -4.2725 | 0.2954 | <.0001 | -0.0001 | 0.0004 | 0.8049 | -0.0015 | 0.0008 | 0.073 | -0.00003 | 0.00002 | 0.1108 |
|  | Intervention | -0.0188 | 0.0108 | 0.0814 | 5.0731 | 2.8573 | 0.0758 | 0.0067 | 0.0040 | 0.0938 | -0.1188 | 0.0075 | <.0001 | -0.0004 | 0.0002 | 0.015 |
|  | Time after intervention | -0.0073 | 0.0100 | 0.4664 | -15.3574 | 2.6359 | <.0001 | -0.0117 | 0.0037 | 0.0015 | -0.1090 | 0.0072 | <.0001 | -0.0005 | 0.0002 | 0.0065 |
| Bipolar | Age |  |  |  |  |  |  |  |  |  |  |  |  |  |  |  |
| Disorder | <20y | Ref. |  |  | Ref. |  |  | Ref. |  |  | Ref. |  |  | Ref. |  |  |
|  | 20-44y | -0.0607 | 0.0163 | 0.0002 | 9.5451 | 2.9229 | 0.0011 | -0.0949 | 0.0103 | <.0001 | -0.5680 | 0.0135 | <.0001 | -0.0036 | 0.0004 | <.0001 |
|  | 45-64y | -0.1413 | 0.0166 | <.0001 | 36.6318 | 2.9838 | <.0001 | -0.2071 | 0.0105 | <.0001 | -1.0681 | 0.0136 | <.0001 | -0.0071 | 0.0004 | <.0001 |
|  | 65-79y | -0.1992 | 0.0192 | <.0001 | 58.3166 | 3.4379 | <.0001 | -0.2601 | 0.0121 | <.0001 | -1.3521 | 0.0147 | <.0001 | -0.0084 | 0.0004 | <.0001 |
|  | ≥80y | -0.2342 | 0.0278 | <.0001 | 64.6118 | 4.9866 | <.0001 | -0.3092 | 0.0175 | <.0001 | -1.4653 | 0.0175 | <.0001 | -0.0089 | 0.0005 | <.0001 |
|  | Health Insurance type |  |  |  |  |  |  |  |  |  |  |  |  |  |  |  |
|  | Health Insurance | Ref. |  |  | Ref. |  |  | Ref. |  |  | Ref. |  |  | Ref. |  |  |
|  | Medical aid | -0.0066 | 0.0085 | 0.4372 | 60.4161 | 1.5297 | <.0001 | -0.1403 | 0.0054 | <.0001 | 0.6253 | 0.0070 | <.0001 | 0.0004 | 0.0002 | 0.0258 |
|  | Sex |  |  |  |  |  |  |  |  |  |  |  |  |  |  |  |
|  | Male | Ref. |  |  | Ref. |  |  | Ref. |  |  | Ref. |  |  | Ref. |  |  |
|  | Female | -0.0435 | 0.0075 | <.0001 | -25.4569 | 1.3392 | <.0001 | 0.0527 | 0.0047 | <.0001 | 0.2721 | 0.0051 | <.0001 | 0.0014 | 0.0001 | <.0001 |
|  | Time | 0.0026 | 0.0019 | 0.1585 | -0.5418 | 0.3360 | 0.1069 | 0.0030 | 0.0012 | 0.0102 | 0.0006 | 0.0013 | 0.6408 | -0.0001 | 0.0000 | 0.0415 |
|  | Intervention | -0.0415 | 0.0178 | 0.0196 | -4.5949 | 3.1868 | 0.1494 | -0.0019 | 0.0112 | 0.8638 | -0.1252 | 0.0115 | <.0001 | -0.0009 | 0.0003 | 0.0056 |
|  | Time after intervention | -0.0341 | 0.0163 | 0.0371 | -6.9793 | 2.9336 | 0.0174 | -0.0179 | 0.0103 | 0.0825 | -0.0792 | 0.0112 | <.0001 | -0.0009 | 0.0003 | 0.0063 |
| Depression | Age |  |  |  |  |  |  |  |  |  |  |  |  |  |  |  |
|  | <20y | Ref. |  |  | Ref. |  |  | Ref. |  |  | Ref. |  |  | Ref. |  |  |
|  | 20-44y | -0.1423 | 0.0081 | <.0001 | 0.6354 | 1.3651 | 0.6416 | -0.0189 | 0.0056 | 0.0008 | -0.3215 | 0.0046 | <.0001 | -0.0028 | 0.0001 | <.0001 |
|  | 45-64y | -0.1772 | 0.0081 | <.0001 | 19.0953 | 1.3713 | <.0001 | -0.1425 | 0.0056 | <.0001 | -0.9081 | 0.0045 | <.0001 | -0.0040 | 0.0001 | <.0001 |
|  | 65-79y | -0.2046 | 0.0088 | <.0001 | 29.2662 | 1.4847 | <.0001 | -0.2136 | 0.0061 | <.0001 | -1.2129 | 0.0047 | <.0001 | -0.0046 | 0.0001 | <.0001 |
|  | ≥80y | -0.1980 | 0.0107 | <.0001 | 67.9726 | 1.8131 | <.0001 | -0.2573 | 0.0075 | <.0001 | -1.4114 | 0.0054 | <.0001 | -0.0045 | 0.0001 | <.0001 |
|  | Health Insurance type |  |  |  |  |  |  |  |  |  |  |  |  |  |  |  |
|  | Health Insurance | Ref. |  |  | Ref. |  |  | Ref. |  |  | Ref. |  |  | Ref. |  |  |
|  | Medical aid | 0.0429 | 0.0057 | <.0001 | 47.7102 | 0.9716 | <.0001 | -0.1136 | 0.0040 | <.0001 | 0.6653 | 0.0028 | <.0001 | 0.0010 | 0.0001 | <.0001 |
|  | Sex |  |  |  |  |  |  |  |  |  |  |  |  |  |  |  |
|  | Male | Ref. |  |  | Ref. |  |  | Ref. |  |  | Ref. |  |  | Ref. |  |  |
|  | Female | -0.0503 | 0.0048 | <.0001 | -15.6578 | 0.8171 | <.0001 | 0.0805 | 0.0034 | <.0001 | 0.1249 | 0.0019 | <.0001 | 0.0004 | 0.00004 | <.0001 |
|  | Time | 0.0035 | 0.0012 | 0.0026 | -0.3191 | 0.1975 | 0.1062 | 0.0038 | 0.0008 | <.0001 | 0.0138 | 0.0005 | <.0001 | -0.0001 | 0.00001 | <.0001 |
|  | Intervention | -0.0230 | 0.0111 | 0.0381 | -3.5132 | 1.8767 | 0.0612 | 0.0081 | 0.0077 | 0.2949 | -0.0958 | 0.0042 | <.0001 | -0.0004 | 0.0001 | <.0001 |
|  | Time after intervention | -0.0374 | 0.0101 | 0.0002 | -6.8650 | 1.7140 | <.0001 | -0.0140 | 0.0071 | 0.0466 | -0.0783 | 0.0040 | <.0001 | -0.0003 | 0.0001 | 0.0003 |
| Anxiety | Age |  |  |  |  |  |  |  |  |  |  |  |  |  |  |  |
| disorder | <20y | Ref. |  |  | Ref. |  |  | Ref. |  |  | Ref. |  |  | Ref. |  |  |
|  | 20-44y | -0.1407 | 0.0130 | <.0001 | -1.0542 | 2.3307 | 0.651 | 0.0321 | 0.0126 | 0.0107 | -0.2432 | 0.0066 | <.0001 | -0.0034 | 0.0003 | <.0001 |
|  | 45-64y | -0.1825 | 0.0129 | <.0001 | 4.4014 | 2.3173 | 0.0575 | -0.1062 | 0.0125 | <.0001 | -0.7510 | 0.0065 | <.0001 | -0.0057 | 0.0003 | <.0001 |
|  | 65-79y | -0.1700 | 0.0137 | <.0001 | 14.6728 | 2.4449 | <.0001 | -0.2315 | 0.0132 | <.0001 | -0.9116 | 0.0067 | <.0001 | -0.0079 | 0.0003 | <.0001 |
|  | ≥80y | -0.1988 | 0.0150 | <.0001 | 58.6192 | 2.6907 | <.0001 | -0.3280 | 0.0145 | <.0001 | -0.9954 | 0.0075 | <.0001 | -0.0077 | 0.0003 | <.0001 |
|  | Health Insurance type |  |  |  |  |  |  |  |  |  |  |  |  |  |  |  |
|  | Health Insurance | Ref. |  |  | Ref. |  |  | Ref. |  |  | Ref. |  |  | Ref. |  |  |
|  | Medical aid | 0.0635 | 0.0083 | <.0001 | 51.2559 | 1.4788 | <.0001 | -0.1591 | 0.0080 | <.0001 | 0.7739 | 0.0042 | <.0001 | 0.0034 | 0.0002 | <.0001 |
|  | Sex |  |  |  |  |  |  |  |  |  |  |  |  |  |  |  |
|  | Male | Ref. |  |  | Ref. |  |  | Ref. |  |  | Ref. |  |  | Ref. |  |  |
|  | Female | -0.0462 | 0.0059 | <.0001 | -7.9807 | 1.0559 | <.0001 | 0.0557 | 0.0057 | <.0001 | 0.1121 | 0.0021 | <.0001 | 0.0011 | 0.0001 | <.0001 |
|  | Time | 0.0018 | 0.0014 | 0.2249 | -0.6954 | 0.2590 | 0.0073 | 0.0006 | 0.0014 | 0.6912 | 0.0120 | 0.0005 | <.0001 | -0.0003 | 0.00002 | <.0001 |
|  | Intervention | -0.0255 | 0.0141 | 0.0708 | -0.6046 | 2.5247 | 0.8107 | -0.0142 | 0.0136 | 0.2973 | -0.0730 | 0.0048 | <.0001 | -0.0006 | 0.0002 | 0.0007 |
|  | Time after intervention | -0.0059 | 0.0126 | 0.6381 | -2.3786 | 2.2509 | 0.2907 | -0.0074 | 0.0121 | 0.5408 | -0.0334 | 0.0046 | <.0001 | -0.0002 | 0.0002 | 0.3978 |
| Other mental | Age |  |  |  |  |  |  |  |  |  |  |  |  |  |  |  |
| disorders | <20y | Ref. |  |  | Ref. |  |  | Ref. |  |  | Ref. |  |  | Ref. |  |  |
|  | 20-44y | -0.1257 | 0.0419 | 0.0027 | 35.1474 | 14.8892 | 0.0183 | -0.0303 | 0.0247 | 0.2213 | -0.5114 | 0.0282 | <.0001 | -0.0019 | 0.0004 | <.0001 |
|  | 45-64y | -0.1825 | 0.0406 | <.0001 | 45.1581 | 14.4252 | 0.0017 | -0.1238 | 0.0240 | <.0001 | -1.1019 | 0.0270 | <.0001 | -0.0044 | 0.0004 | <.0001 |
|  | 65-79y | -0.2191 | 0.0404 | <.0001 | 8.0852 | 14.3671 | 0.5736 | -0.1194 | 0.0239 | <.0001 | -1.0273 | 0.0270 | <.0001 | -0.0046 | 0.0004 | <.0001 |
|  | ≥80y | -0.1857 | 0.0405 | <.0001 | 40.6340 | 14.3882 | 0.0047 | -0.1382 | 0.0239 | <.0001 | -0.9280 | 0.0270 | <.0001 | -0.0044 | 0.0004 | <.0001 |
|  | Health Insurance type |  |  |  |  |  |  |  |  |  |  |  |  |  |  |  |
|  | Health Insurance | Ref. |  |  | Ref. |  |  | Ref. |  |  | Ref. |  |  | Ref. |  |  |
|  | Medical aid | 0.0182 | 0.0089 | 0.0417 | 80.3349 | 3.1820 | <.0001 | -0.0499 | 0.0053 | <.0001 | 0.5030 | 0.0037 | <.0001 | 0.0004 | 0.0001 | <.0001 |
|  | Sex |  |  |  |  |  |  |  |  |  |  |  |  |  |  |  |
|  | Male | Ref. |  |  | Ref. |  |  | Ref. |  |  | Ref. |  |  | Ref. |  |  |
|  | Female | -0.0202 | 0.0080 | 0.0113 | -29.7967 | 2.8348 | <.0001 | -0.0030 | 0.0047 | 0.5214 | -0.0406 | 0.0023 | <.0001 | -0.0001 | 0.00003 | <.0001 |
|  | Time | 0.0020 | 0.0019 | 0.2764 | -1.4020 | 0.6660 | 0.0353 | 0.0012 | 0.0011 | 0.2917 | -0.0022 | 0.0006 | 0.0001 | -0.00002 | 0.00001 | 0.0044 |
|  | Intervention | 0.0213 | 0.0199 | 0.2859 | 13.5720 | 7.0874 | 0.0555 | 0.0148 | 0.0118 | 0.21 | -0.0319 | 0.0049 | <.0001 | -0.000002 | 0.0001 | 0.9793 |
|  | Time after intervention | -0.0281 | 0.0170 | 0.0981 | -15.1996 | 6.0489 | 0.012 | 0.0051 | 0.0101 | 0.6091 | -0.0701 | 0.0047 | <.0001 | 0.00002 | 0.0001 | 0.7356 |
| Sleep disorder | Age |  |  |  |  |  |  |  |  |  |  |  |  |  |  |  |
|  | <20y | Ref. |  |  | Ref. |  |  | Ref. |  |  | Ref. |  |  | Ref. |  |  |
|  | 20-44y | 0.0426 | 0.0442 | 0.3357 | 12.1996 | 11.1245 | 0.2728 | -0.1181 | 0.0372 | 0.0015 | 0.4743 | 0.0177 | <.0001 | -0.0019 | 0.0003 | <.0001 |
|  | 45-64y | 0.0132 | 0.0437 | 0.7615 | 14.0891 | 10.9742 | 0.1992 | -0.2147 | 0.0367 | <.0001 | 0.1778 | 0.0176 | <.0001 | -0.0023 | 0.0003 | <.0001 |
|  | 65-79y | 0.0316 | 0.0440 | 0.4718 | 27.6079 | 11.0540 | 0.0125 | -0.2573 | 0.0369 | <.0001 | 0.2012 | 0.0176 | <.0001 | -0.0027 | 0.0003 | <.0001 |
|  | ≥80y | -0.0279 | 0.0444 | 0.53 | 75.3974 | 11.1698 | <.0001 | -0.2854 | 0.0373 | <.0001 | 0.2577 | 0.0180 | <.0001 | -0.0027 | 0.0003 | <.0001 |
|  | Health Insurance type |  |  |  |  |  |  |  |  |  |  |  |  |  |  |  |
|  | Health Insurance | Ref. |  |  | Ref. |  |  | Ref. |  |  | Ref. |  |  | Ref. |  |  |
|  | Medical aid | 0.0505 | 0.0121 | <.0001 | 55.7088 | 3.0455 | <.0001 | -0.1069 | 0.0102 | <.0001 | 0.7178 | 0.0052 | <.0001 | 0.0011 | 0.0001 | <.0001 |
|  | Sex |  |  |  |  |  |  |  |  |  |  |  |  |  |  |  |
|  | Male | Ref. |  |  | Ref. |  |  | Ref. |  |  | Ref. |  |  | Ref. |  |  |
|  | Female | -0.0790 | 0.0098 | <.0001 | 0.4918 | 2.4737 | 0.8424 | 0.0542 | 0.0083 | <.0001 | 0.0447 | 0.0028 | <.0001 | -0.0002 | 0.00004 | <.0001 |
|  | Time | -0.0039 | 0.0024 | 0.1047 | -1.3674 | 0.6117 | 0.0254 | 0.0026 | 0.0020 | 0.2121 | 0.0086 | 0.0007 | <.0001 | -0.00004 | 0.00001 | 0.0002 |
|  | Intervention | 0.0110 | 0.0236 | 0.6404 | 1.1060 | 5.9441 | 0.8524 | -0.0265 | 0.0199 | 0.1827 | -0.0631 | 0.0062 | <.0001 | -0.0002 | 0.0001 | 0.0369 |
|  | Time after intervention | 0.0255 | 0.0211 | 0.2273 | -2.3022 | 5.3096 | 0.6646 | -0.0181 | 0.0177 | 0.3073 | -0.0043 | 0.0061 | 0.4830 | -0.0001 | 0.0001 | 0.3048 |
| Others | Age |  |  |  |  |  |  |  |  |  |  |  |  |  |  |  |
|  | <20y | Ref. |  |  | Ref. |  |  | Ref. |  |  | Ref. |  |  | Ref. |  |  |
|  | 20-44y | -0.2375 | 0.0073 | <.0001 | 25.0958 | 1.5921 | <.0001 | -0.0864 | 0.0042 | <.0001 | -0.1780 | 0.0031 | <.0001 | 0.0076 | 0.0001 | <.0001 |
|  | 45-64y | -0.2949 | 0.0070 | <.0001 | 29.4193 | 1.5326 | <.0001 | -0.1347 | 0.0041 | <.0001 | -0.6259 | 0.0032 | <.0001 | 0.0116 | 0.0001 | <.0001 |
|  | 65-79y | -0.3758 | 0.0080 | <.0001 | 49.4469 | 1.7308 | <.0001 | -0.1093 | 0.0046 | <.0001 | -0.7868 | 0.0039 | <.0001 | 0.0050 | 0.0002 | <.0001 |
|  | ≥80y | -0.3414 | 0.0105 | <.0001 | 31.1638 | 2.2887 | <.0001 | -0.0096 | 0.0061 | 0.1152 | -0.8557 | 0.0062 | <.0001 | 0.0051 | 0.0003 | <.0001 |
|  | Health Insurance type |  |  |  |  |  |  |  |  |  |  |  |  |  |  |  |
|  | Health Insurance | Ref. |  |  | Ref. |  |  | Ref. |  |  | Ref. |  |  | Ref. |  |  |
|  | Medical aid | 0.0734 | 0.0035 | <.0001 | 69.4998 | 0.7675 | <.0001 | -0.1523 | 0.0020 | <.0001 | 0.5209 | 0.0035 | <.0001 | 0.0039 | 0.0002 | <.0001 |
|  | Sex |  |  |  |  |  |  |  |  |  |  |  |  |  |  |  |
|  | Male | Ref. |  |  | Ref. |  |  | Ref. |  |  | Ref. |  |  | Ref. |  |  |
|  | Female | -0.0822 | 0.0040 | <.0001 | -28.4083 | 0.8606 | <.0001 | 0.1771 | 0.0023 | <.0001 | 0.0141 | 0.0024 | <.0001 | -0.0003 | 0.0001 | 0.003 |
|  | Time | -0.0025 | 0.0008 | 0.0019 | -0.1313 | 0.1785 | 0.4620 | 0.0003 | 0.0005 | 0.5282 | 0.0093 | 0.0006 | <.0001 | -0.0003 | 0.00003 | <.0001 |
|  | Intervention | -0.0292 | 0.0081 | 0.0003 | -6.6305 | 1.7524 | 0.0002 | 0.0007 | 0.0046 | 0.8750 | -0.1065 | 0.0053 | <.0001 | -0.0030 | 0.0002 | <.0001 |
|  | Time after intervention | -0.0212 | 0.0073 | 0.0035 | -13.0691 | 1.5788 | <.0001 | -0.0122 | 0.0042 | 0.0035 | -0.0645 | 0.0050 | <.0001 | -0.0040 | 0.0002 | <.0001 |

Abbreviation Est. (Estimate), SE (Standard Error), P (P-value)

Other mental disorders: Other mental disorders due to brain damage and dysfunction and to physical disease, Others: remainder of mental disorder
